# Progress and prospects in antisense oligonucleotide-mediated exon skipping therapies for Duchenne muscular dystrophy

**DOI:** 10.1007/s10974-024-09688-2

**Published:** 2025-01-30

**Authors:** Katarzyna Chwalenia, Matthew J.A. Wood, Thomas C. Roberts

**Affiliations:** 1 Institute of Developmental and Regenerative Medicine, University of Oxford, IMS-Tetsuya Nakamura Building, Old Road Campus, Roosevelt Dr, Headington, Oxford, OX3 7TY, UK; 2 Department of Paediatrics, University of Oxford, South Parks Road, Oxford, OX1 3QX, UK; 3 MDUK Oxford Neuromuscular Centre, Oxford, UK, OX3 7TY, UK

## Abstract

Recent years have seen enormous progress in the field of advanced therapeutics for the progressive muscle wasting disease Duchenne muscular dystrophy (DMD). In particular, four antisense oligonucleotide (ASO) therapies targeting various DMD-causing mutations have achieved FDA approval, marking major milestones in the treatment of this disease. These compounds are designed to induce alternative splicing events that restore the translation reading frame of the dystrophin gene, leading to the generation of internally-deleted, but mostly functional, pseudodystrophin proteins with the potential to compensate for the genetic loss of dystrophin. However, the efficacy of these compounds is very limited, with delivery remaining a key obstacle to effective therapy. There is therefore an urgent need for improved ASO technologies with better efficacy, and with applicability to a wider range of patient mutations. Here we discuss recent developments in ASO therapies for DMD, and future prospects with a focus on ASO chemical modification and bioconjugation strategies.

## Dystrophinopathy

Duchenne muscular dystrophy is a severe, X-linked inherited disorder caused by loss of the dystrophin protein affecting 1:3,500-5,000 boys worldwide.[1] The disease results from a spectrum of loss-of-function mutations in the dystrophin gene (*DMD*),[1] the most common of which are whole exon deletions.[2] *DMD* is one of the largest genes of the human genome, encoding a 14 kb, 79-exon long mRNA which is translated into a 427 kDa dystrophin protein. In DMD, dystrophin deficiency results in vulnerability of muscles to contraction-induced damage.[3] As such, daily physical function initiates a detrimental cascade of degeneration-regeneration cycles ultimately leading to muscle wasting.[4] Notably, the disease affects not only skeletal muscle, but also the heart, and almost all DMD patients develop a form of dilated cardiomyopathy, often leading to end-stage heart failure.[5] Currently, cardiac dysfunction is the main cause of death for dystrophic patients, typically in the third decade of life.[6]

Becker muscular dystrophy is an allelic form of DMD characterised by later onset, milder disease severity, and slower disease progression with many patients remaining ambulant well into adulthood (with some never requiring wheelchair use).[7–9] In most cases, the difference between the DMD and BMD phenotypes can be explained by the ‘reading frame rule’.[10,11] Specifically, DMD is caused by frameshift mutations which lead to production of defective, prematurely terminated protein variants. Conversely, BMD patients typically maintain the translation reading frame, producing an internally deleted, yet partially functional, pseudodystrophin, which underlies the milder disease progression. A remarkable example of this phenomenon is the case of a BMD patient lacking 46% of the dystrophin gene who presented with only mild symptoms and was ambulant at the age of 61.[7]

## Antisense Oligonucleotide Therapeutics

Antisense nucleotides (ASOs) are short, single-stranded nucleic acid polymers (~20-30 nucleotides in length) designed to bind to complementary target mRNA sequences via Watson-Crick base pairing.[12–15] The therapeutic potential of ASOs was first demonstrated in 1978 by Stephenson and Zamecnik who showed that short, synthetic DNA oligonucleotides can inhibit replication of the Rous sarcoma virus material in a cell-free system.[16] These results have paved the way for research into short oligonucleotide-based therapeutics, and eventually to the regulatory approval of ~15 ASO-based drugs over the past 26 years, the vast majority within the last decade.[17] ASOs elicit gene regulatory functions via a variety of mechanisms including post-transcriptional control through transcript degradation, translation inhibition, or splice-switching [17,18]. In the context of DMD, ASO-mediated exon skipping of the dystrophin pre-mRNA is one of the most promising therapeutic strategies.[12] This approach aims to convert the severe DMD phenotype into a less severe ‘BMD-like’ phenotype by paradoxically increasing the degree of internal deletion and restoring the translation reading frame in the dystrophin protein via the exclusion of a specific exon(s) from the *DMD* pre-mRNA. This leads to the production of an internally-deleted but largely functional pseudodystrophin protein.[12,19] Importantly, ASO-mediated exon skipping is a mutation-specific approach, theoretically applicable to as many as ~83% of all DMD-causing mutations.[19,20]

Due to their inherent susceptibility to nucleases and poor target binding affinity, unmodified ASOs based on natural DNA and RNA chemistry have limited clinical utility.[14] As such, diverse ASO chemistries have been developed to improve their ‘drug-likeness’.[14] The most commonly used nucleic acid chemistry in DMD exon skipping therapeutics is PMO (phosphorodiamidate morpholino oligonucleotide). PMOs consist of a six-membered morpholine ring (analogous to the five-membered arabinose ring found in native DNA/RNA) and an uncharged phosphorodiamidate backbone (analogous to the phosphodiester linkages). The non-natural chemical composition of PMOs means that these molecules cannot be degraded by biofluid or tissue nucleases, and as such these molecules exhibit high stability and a very favourable safety profile.[21,22] Indeed, PMOs have been administered to rodents at doses of up to 3g/kg [23]. As of 2024, four PMO antisense drugs have been approved by the FDA for treatment of DMD (Table 1): eteplirsen, golodirsen, viltolarsen and casimersen.[24–28]

Eteplirsen (developed by Sarepta Therapeutics) was the first drug to receive marketing authorisation in 2016[24], which marked a significant milestone in the development of therapeutics for DMD [24]. This was the first gene-specific drug offered to DMD patients, which utilised a novel, targeted mechanism as opposed to the pleiotropic mode of action of corticosteroids, which are the standard-of-care in DMD management.[29]. The clinical trials leading to the eteplirsen approval begun in 2007 with a first phase 1/2 study conducted at Imperial College London demonstrating the safety and tolerability of the drug after intramuscular injection in patients (NCT00159250).[30] A further, phase 2, dose escalation, intravenous injection trial has proven the safety profile of eteplirsen, with no drug-related serious adverse events reported in the cohort of 19 ambulant DMD patients (NCT00844597).[31] The exon-skipping efficacy and thus therapeutic benefit of the drug were variable in these studies with a maximum increase of 16% in dystrophin levels as measured by western blot in the phase 2 trial.[31]. Follow-up studies reported more modest findings. Treatment with weekly injections of eteplirsen for almost 3.5 years resulted in the restoration of ~1% of dystrophin levels observed in healthy muscle [24]. As such, the approval of eteplirsen by FDA in 2016 was highly controversial, as it was solely based on the minimal dystrophin restoration as a surrogate endpoint, without the evidence of functional benefit.[32]. Similarly modest results were obtained with other approved ASOs (Table 1).[24–28,33–35] For example, dystrophin restoration levels were the highest for viltolarsen with 5.9% of dystrophin expression achieved after 25 weeks of treatment.[26] Clinical trials for all of the FDA-approved exon skipping ASOs are currently ongoing.[36] Notably, none of the FDA-approved ASOs for DMD have been approved by European Medicine Agency, based on the same efficacy data.[32]

## Enhancing ASO Activity

Due to the limited efficacy of available drugs, the pursuit of improved ASO therapeutics in DMD continues. Here we will consider the two major approaches for ASO improvement; (i) chemical modification, and (ii) bioconjugation.

A major development in oligonucleotide chemistry is the incorporation of stereopure backbone linkages. While phosphodiester backbones are achiral, the incorporation of a sulphur to replace one of the non-bridging backbone oxygen atoms, as in the case of phosphorothioate modification gives rise to chiral centres at every backbone linkage position. Control of the stereochemistry at each position has been reported to improve many oligonucleotide properties, with a stereopure enantiomer ultimately being more potent than the racemic bulk mixture.[37] To this end, Wave Life Sciences developed a stereopure ASO with phosphorothioate backbone to induce *DMD* exon 51 skipping (suvodirsen).[38] Despite promising pre-clinical results, suvodirsen failed to meet the primary endpoint (i.e. an increase in dystrophin protein expression) in a phase I clinical trial (NCT03508947), and its development was subsequently discontinued.[39]. However, Wave Life Science have pursued a second seteropure ASO designed to skip *DMD* exon 53 (WVE-N531). This compound is a mixmer ASO containing 2’-*O*-methyl and 2’-fluoro sugar modifications, and a mixture of stereoselective phosphorothioate and phosphoryl guanidine (PN) backbone linkages.[40] This pattern of ASO chemistry has been shown to improve the delivery and overall therapeutic potency in animal models, including the severely affected dystrophin/utrophin double knock-out (dKO) mouse model of DMD.[37,41] WVE-N531 is currently being evaluated in a phase 1b/2 trial (FORWARD-53, NCT04906460). Initial reports from this trial have been highly encouraging, with a mean level of exon skipping of 53% after three biweekly 10 mg/kg doses in the first three DMD patients treated, although dystrophin protein levels were negligible [41]. Interim data from this trial after 24 weeks of dosing recently reported mean dystrophin protein expression levels of 9% (muscle content-adjusted) and 5.5% (unadjusted), with mean exon skipping levels of 57%.[42]

Notably, a 2’-*O*-methyl ASO with phosphorothioate backbone, developed by BioMarin (drisapersen) was ultimately discontinued after demonstrating limited efficacy (i.e. lack of functional improvement) and adverse events including injection site reactions, thrombocytopenia, and proteinuria in a phase 3 trial (NCT01254019).[43,44] The incorporation of phosphorothioate linkages was associated with dose limiting renal toxicity, which may prove to be a challenge for other therapeutic modalities, given the widespread usage of this chemistry in the nucleic acid therapeutic field. Nevertheless, other phosphorothioate containing ASOs are in development at present. Daiichi Sankyo is developing renadirsen (DS 5141b) a fully phosphorothioate mixmer containing 2’-*O*-methyl and ethylene bridged nucleic acid (ENA) modifications for the skipping of *DMD* exon 45,[45] currently under investigation in a phase 1/2 clinical trial (NCT04433234). Furthermore, SQY Therapeutics is investigating a completely distinct chemistry, tricyclo-DNA. An ASO consisting of this chemistry, and conjugated to a palmitic acid, (SQY51) designed to skip *DMD* exon 51 is currently under investigation in a phase ½ clinical trial (NCT05753462/AVANCE1). Tricyclo-DNA is notable, as it has been reported to enable exon skipping in the brain, meaning that the neurological pathologies of DMD may be treatable.[46,47]

A key advantage of FDA-approved PMO chemistry is the favourable safety profile resulting from their low plasma protein binding properties,[14,15] although this same characteristic also leads to rapid plasma clearance of the PMO via the kidneys.[14,15,36,48,49] This results not only in the requirement for weekly dosing for prolonged periods, but also in limited uptake by targeted cells. As such, the efficient delivery of PMO to the muscle (and especially heart) remains an ongoing challenge for the field. An alternative approach for ASO improvement is via covalent conjugation to a delivery-assisting moiety. This has been most extensively studied in the case of PMOs, whereby the uncharged backbone enables facile synthesis of a variety of conjugates.

One of the most promising technologies for improved tissue targeting and uptake is conjugation of the PMO with cell-penetrating peptides (CPPs).[48,49] CPPs are short (<30 amino acids), often cationic, sequences that possess the remarkable ability to cross cell membranes, making them valuable tools for delivering therapeutic molecules.[50] A plethora of peptide-conjugated PMOs (PPMOs) has been developed and pre-clinically tested in the context of DMD.[48] In comparison to unconjugated PMOs, PPMOs demonstrate enhanced efficacy as splicing correctors at lower doses, enabling systemic administration to achieve widespread dystrophin restoration in skeletal muscles and cardiac tissue [48]. Multiple recent clinical trials of PPMOs for DMD are ongoing or have recently completed: Sarepta (NCT04004065/MOMENTUM, SRP-5051/vesleteplirsen), PepGen (NCT06079736, PGN-EDO51) and Entrada (ENTR-601-44-101, ENTR-601-44).[12] The interim phase 2 data of vesleteplirsen (which consists of a PMO conjugated to the R_6_Gly peptide) trial revealed mean 11% of exon skipping and 5.7% of dystrophin expression after 7 monthly doses.[51] However, in November 2024, Sarepta announced the discontinuation of its vesleteplirsen programme on account of prolonged hypomagnesemia and a decline in the renal function marker eGFR (estimated glomerular filtration rate) in subsets of participants in the MOMENTUM trial.[52,53]

In the PepGen trial, a single PGN-EDO51 dose resulted in 2% of exon skipping in healthy volunteers.[54] Interestingly, PepGen also observed transient hypomagnesemia in two PPMO-treated individuals, which resolved without intervention.[55] In contrast with the relatively simple amino acid composition of vesleteplirsen, the PGN-EDO51 is based on extensive iterative improvements of the Pip (PMO internalisation peptide) series of peptides developed through a collaboration between the groups of Mike Gait and Matthew Wood.[49,56–58] The latest versions of these peptides have been optimised to minimise renal toxicity without compromising exon skipping activity. It remains to be demonstrated whether PGN-EDO51 remains safe after prolonged treatment. Similarly, the PPMO compounds of Entrada are based on a cyclic peptide design, although more detailed chemical details of the structures of these compounds is not publicly available. Preliminary data from the phase 1 trial of ENTR-601-44 targeting *DMD* exon 44 (ENTR-601-44-101) in healthy volunteers suggested that the drug is safe, with exon skipping levels of up to 0.65% reported.[59] A phase 2 trial in amenable DMD patients is expected to be initiated in 2025.

Several PMO-bioconjugation strategies have been explored which target the ubiquitously-expressed transferrin receptor 1 (TFRC, Trf1), in order to promote uptake in skeletal and cardiac muscle tissues.[12] Avidity Biosciences is developing antibody oligonucleotide conjugates (AOCs) consisting of a monoclonal antibody targeting TFRC and a PMO designed to skip *DMD* exon 44 (i.e. Delpacibart zotadirseb, del-zota, or AOC 1044). This compound is currently under investigation in a phase 1/2 clinical trial (NCT05670730/EXPLORE44) with interim findings showing exon skipping levels of 1.5% after a single 10 mg/kg dose in healthy volunteers.[60]

Similarly, Dyne Therapeutics are developing DYNE-251, a Fab fragment targeting TFRC conjugated to a PMO designed to skip *DMD* exon 51. Dyne has recently reported positive preclinical findings from their platform technology (known as FORCE).[61] DYNE-251 is currently under investigation in a phase 1/2 clinical trial (NCT05524883/DELIVER) with interim data showing that patients treated at the 20 mg/kg dose exhibited 8.7% of healthy dystrophin levels (muscle content adjusted) and 3.7% (unadjusted) and functional improvements at 6 months post treatment for patients treated with both 20 mg/kg and 10 mg/kg monthly doses.[62,63]

A discrepancy between RNA and protein levels has been a common observation across clinical programmes.[25,41] For example, viltolarsen induced an 43.9% increase in exon-skipping which resulted only in production of 5.9% of the wild-type dystrophin levels [25]. It is well known that RNA and protein levels correlate poorly in general,[64,65] although for genetic therapies a close relationship is often taken for granted. However, it is important to note that such inconsistences between levels of corrected transcripts and restored dystrophin protein may a consequence of technical issues related to assay design, the timing of sample collection (i.e. there potentially being a lag between RNA level correction and the accumulation of dystrophin protein), differences in protein stability when comparing full-length healthy dystrophin with internally-deleted pseudodystrophins restored by exon skipping, or potentially other pathobiological features which may cause an obstacle to therapeutic success that are as yet under-appreciated (e.g. post-transcriptional repression of dystrophin expression via trans-acting factors in dystrophic muscle).

## Competition from microdystrophin gene therapy

Up until 2023, ASO-based drugs were the only option to treat the underlying cause of DMD. This changed with the approval of the delandistrogene moxeparvovec (elevidys, SRP-9001), an AAV-based gene replacement therapy developed by Sarepta Therapeutics.[66] This drug encodes a minigene version of dystrophin, known as microdystrophin, in which large regions of the dystrophin open reading frame have been deleted in order to be small enough to be packaged into an AAV particle.[12] A phase 2 trial of elevidys demonstrated restoration of almost 40% of microdystrophin protein levels in patients at week 12 post-infusion (assessed by western blot).[67] Despite relatively high levels of microdystrophin, improvements in clinical endpoints have so far been very modest. The strongest evidence for any benefit was in the 5-6 year age group, but the effect was much less apparent older boys.[67] This raises the important question of whether microdystrophin(s), even when expressed at relatively high levels, are capable of replacing sufficient dystrophin function to allow meaningful clinical benefit.

Notably, in a parallel microdystrophin study sponsored by Genethon, investigators observed up to 85% of dystrophin positive myofibres by immunofluorescence 8-weeks post injection.[68] However, the gene replacement approach is likely to result in diminished pseudodystrophin production with time due to vector genome loss, epigenetic silencing of the transgene, and the dilution effect of non-transduced nuclei as a consequence of muscle growth and repair. Notably, repeat administration with AAV is currently challenging as the patient is effectively immunised to the treatment after the first dose.[67] Moreover, AAV poses a substantial safety risk with some clinical trials previously reporting fatal immune adverse effects using high doses of the viral vectors (including with the Pfizer microdystrophin).[69] As such, the search for efficacious and safe DMD therapeutics is far from over, with ASOs, gene therapy, utrophin (a natural dystrophin paralogue) upregulation,[70], and CRISPR-Cas9[71–73] all showing promise.[12] While most of these strategies mimic dystrophin protein (gene replacement) and target the dystrophin mRNA (ASOs) or gene (CRISPR-Cas9), numerous aspects of dystrophin biology remain underexplored and poorly understood. For example, relatively little is known about the specific mechanisms governing correct dystrophin localisation to the sarcolemma. For example, our group has shown that the pattern of dystrophin coverage across the sarcolemma is an important consideration for effective restoration of muscle integrity,[74] and that various therapeutic interventions restore dystrophin with distinct distribution patterns.[74] Specifically, ASO-mediated exon skipping induced a uniform pattern of dystrophin distribution, whereas CRISPR-Cas9-mediated exon deletion resulted in a patchy pattern of dystrophin at the sarcolemma.[58,72,75] As such, ASO therapy may present an advantage over other modalities in some cases, in terms of the uniformity of sarcolemmal dystrophin coverage post treatment.

## Conclusions

In conclusion, while great progress has been made in the field of ASO-mediated exon skipping therapies for DMD, there is still a need for better drug delivery and improved efficacy. These challenges may be addressed through the use of novel ASO chemical modifications and bioconjugation.

In this special *festschrift* issue, we recognise the career achievements of Professor Jenny Morgan. While Prof Morgan’s contributions to the field of satellite cell biology and myoblast transfer are widely recognised as world leading,[76–80] she has also made key contributions to the field of ASO-mediated exon skipping. Prof Morgan was a founding member of the MDEX consortium, which was instrumental in the development of eteplirsen, and was a major contributor and co-author on the first studies to demonstrate dystrophin exon skipping in human DMD patient muscle. [30,31] In addition, she has made numerous important contributions to this field, including development of a standardised method to assess exon-skipping in eteplirsen-treated patients and, more recently, in relation to the clinical evaluation of the exon 53 skipping compound golodirsen.[81,82] An immortalised myoblast cell line derived from the *mdx* mouse (H2K-*mdx*) developed by Prof Morgan has become a widely-used cell model for screening exon skipping compounds that is still in use today.[83] Collectively, these contributions have been critical for the clinical realisation of exon skipping as a highly novel DMD therapeutic modality, and form the foundations on which future developments are built.

## Figures and Tables

**Table 1:** FDA-approved ASO exon skipping therapies for DMD.

ASO	*DMD* Target exon	Sequence (5’ to 3’)	Company	FDA Approval	Dystrophin protein restoration	Reference
**Eteplirsen**	51	CTCCAACATCAAGGAAGATGGCATTTCTAG	Sarepta	Sept 2016	0.9% after 180 weeks	[24]
**Golodirsen**	53	GTTGCCTCCGGTTCTGAAGGTGTTC	Sarepta	Dec 2019	1% after 48 weeks	[27,33]
**Viltolarsen**	53	CCTCCGGTTCTGAAGGTGTTC	NS Pharma	Aug 2020	5.9% after 25 weeks	[25,26]
**Casimersen**	45	CAATGCCATCCTGGAGTTCCTG	Sarepta	Feb 2021	4.25% after 48 weeks	[28,35]

All ASO sequences consist of phosphorodiamidate morpholino oligonucleotide (PMO) chemistry.

## References

[1] Hoffman EP, Brown RH, Kunkel LM (1987). Dystrophin: The protein product of the duchenne muscular dystrophy locus. Cell.

[2] Muntoni F, Torelli S, Ferlini A (2003). Dystrophin and mutations: one gene, several proteins, multiple phenotypes. Lancet Neurol.

[3] Petrof BJ, Shrager JB, Stedman HH (1993). Dystrophin protects the sarcolemma from stresses developed during muscle contraction. Proc Natl Acad Sci U S A.

[4] Cardone N, Taglietti V, Baratto S (2023). Myopathologic trajectory in Duchenne muscular dystrophy (DMD) reveals lack of regeneration due to senescence in satellite cells. Acta Neuropathol Commun.

[5] Szabo SM, Salhany RM, Deighton A (2021). The clinical course of Duchenne muscular dystrophy in the corticosteroid treatment era: a systematic literature review. Orphanet J Rare Dis.

[6] Passamano L, Taglia A, Palladino A (2012). Improvement of survival in Duchenne Muscular Dystrophy: retrospective analysis of 835 patients. Acta Myologica.

[7] England SB, Nicholson LVB, Johnson MA (1990). Very mild muscular dystrophy associated with the deletion of 46% of dystrophin. Nature.

[8] Clemens PR, Niizawa G, Feng J (2020). The CINRG Becker Natural History Study: Baseline characteristics. Muscle Nerve.

[9] Thada PK, Bhandari J, Umapathi KK (2023). Becker Muscular Dystrophy.

[10] Vengalil S, Preethish-Kumar V, Polavarapu K (2017). Duchenne Muscular Dystrophy and Becker Muscular Dystrophy Confirmed by Multiplex Ligation-Dependent Probe Amplification: Genotype-Phenotype Correlation in a Large Cohort. Journal of Clinical Neurology.

[11] Aartsma-Rus A, Van Deutekom JCT, Fokkema IF (2006). Entries in the Leiden Duchenne muscular dystrophy mutation database: An overview of mutation types and paradoxical cases that confirm the reading-frame rule. Muscle Nerve.

[12] Roberts TC, Wood MJA, Davies KE (2023). Therapeutic approaches for Duchenne muscular dystrophy. Nat Rev Drug Discov.

[13] Aartsma-Rus A, Straub V, Hemmings R (2017). Development of Exon Skipping Therapies for Duchenne Muscular Dystrophy: A Critical Review and a Perspective on the Outstanding Issues. Nucleic Acid Ther.

[14] Roberts TC, Langer R, Wood MJA (2020). Advances in oligonucleotide drug delivery. Nature Reviews Drug Discovery.

[15] Brad Wan W, Seth PP (2016). The Medicinal Chemistry of Therapeutic Oligonucleotides. J Med Chem.

[16] Stephenson ML, Zamecnik PC (1978). Inhibition of Rous sarcoma viral RNA translation by a specific oligodeoxyribonucleotide. Proc Natl Acad Sci U S A.

[17] Lauffer MC, van Roon-Mom W, Aartsma-Rus A (2024). Possibilities and limitations of antisense oligonucleotide therapies for the treatment of monogenic disorders. Communications Medicine.

[18] Dhuri K, Bechtold C, Quijano E (2020). Antisense Oligonucleotides: An Emerging Area in Drug Discovery and Development. J Clin Med.

[19] Aartsma-Rus A, Fokkema I, Verschuuren J (2009). Theoretic applicability of antisense-mediated exon skipping for Duchenne muscular dystrophy mutations. Hum Mutat.

[20] Bladen CL, Salgado D, Monges S (2015). The TREAT-NMD DMD Global Database: analysis of more than 7,000 Duchenne muscular dystrophy mutations. Hum Mutat.

[21] Amantana A, Iversen PL (2005). Pharmacokinetics and biodistribution of phosphorodiamidate morpholino antisense oligomers. Curr Opin Pharmacol.

[22] Summerton J, Weller D (1997). Morpholino antisense oligomers: design, preparation, and properties. Antisense Nucleic Acid Drug Dev.

[23] Wu B, Lu P, Benrashid E (2010). Dose-dependent restoration of dystrophin expression in cardiac muscle of dystrophic mice by systemically delivered morpholino. Gene Ther.

[24] Lim KRQ, Maruyama R, Yokota T (2017). Eteplirsen in the treatment of Duchenne muscular dystrophy. Drug Des Devel Ther.

[25] Roshmi RR, Yokota T (2023). Viltolarsen: From Preclinical Studies to FDA Approval. Methods Mol Biol.

[26] Clemens PR, Rao VK, Connolly AM (2020). Safety, Tolerability, and Efficacy of Viltolarsen in Boys With Duchenne Muscular Dystrophy Amenable to Exon 53 Skipping: A Phase 2 Randomized Clinical Trial. JAMA Neurol.

[27] Heo Y-A (2020). Golodirsen: First Approval. Drugs.

[28] Shirley M (2021). Casimersen: First Approval. Drugs.

[29] Bushby K, Finkel R, Birnkrant DJ (2010). Diagnosis and management of Duchenne muscular dystrophy, part 1: diagnosis, and pharmacological and psychosocial management. Lancet Neurol.

[30] Kinali M, Arechavala-Gomeza V, Feng L (2009). Local restoration of dystrophin expression with the morpholino oligomer AVI-4658 in Duchenne muscular dystrophy: a single-blind, placebo-controlled, dose-escalation, proof-of-concept study. Lancet Neurol.

[31] Cirak S, Arechavala-Gomeza V, Guglieri M (2011). Exon skipping and dystrophin restoration in patients with Duchenne muscular dystrophy after systemic phosphorodiamidate morpholino oligomer treatment: an open-label, phase 2, dose-escalation study. Lancet.

[32] Aartsma-Rus A, Goemans N (2019). A Sequel to the Eteplirsen Saga: Eteplirsen Is Approved in the United States but Was Not Approved in Europe. Nucleic Acid Ther.

[33] Servais L, Mercuri E, Straub V (2022). Long-Term Safety and Efficacy Data of Golodirsen in Ambulatory Patients with Duchenne Muscular Dystrophy Amenable to Exon 53 Skipping: A First-in-human, Multicenter, Two-Part, Open-Label, Phase 1/2 Trial. Nucleic Acid Ther.

[34] Komaki H, Nagata T, Saito T (2018). Systemic administration of the antisense oligonucleotide NS-065/NCNP-01 for skipping of exon 53 in patients with Duchenne muscular dystrophy. Sci Transl Med.

[35] Nicolau S, Malhotra J, Kaler M (2024). Increase in Full-Length Dystrophin by Exon Skipping in Duchenne Muscular Dystrophy Patients with Single Exon Duplications: An Open-label Study. J Neuromuscul Dis.

[36] Vincik LY, Dautel AD, Staples AA (2024). Evolving Role of Viltolarsen for Treatment of Duchenne Muscular Dystrophy. Adv Ther.

[37] Kandasamy P, McClorey G, Shimizu M (2022). Control of backbone chemistry and chirality boost oligonucleotide splice switching activity. Nucleic Acids Res.

[38] Wagner K, Cripe L, Eagle M (2019). EP.83Design of a Phase 2/3 randomized controlled trial of suvodirsen (WVE-210201) in patients with Duchenne muscular dystrophy amenable to exon 51 skipping. Neuromuscular Disorders.

[39] Aartsma-Rus A, Corey DR (2020). The 10th Oligonucleotide Therapy Approved: Golodirsen for Duchenne Muscular Dystrophy. https://home.liebertpub.com/nat.

[40] Kandasamy P, Liu Y, Aduda V (2022). Impact of guanidine-containing backbone linkages on stereopure antisense oligonucleotides in the CNS. Nucleic Acids Res.

[41] Tillinger M, Lake S, Servais L (2023). P22 WVE-N531 yields 53% mean exon 53 skipping in skeletal muscle of boys with Duchenne muscular dystrophy (DMD) after three biweekly doses. Neuromuscular Disorders.

[42] Wave Life Sciences Announces Positive Interim Data from FORWARD-53 Clinical Trial Evaluating WVE-N531 in Boys with Duchenne Muscular Dystrophy Amenable to Exon 53 Skipping.

[43] Markati T, De Waele L, Schara-Schmidt U (2021). Lessons Learned from Discontinued Clinical Developments in Duchenne Muscular Dystrophy. Front Pharmacol.

[44] Goemans N, Mercuri E, Belousova E (2018). A randomized placebo-controlled phase 3 trial of an antisense oligonucleotide, drisapersen, in Duchenne muscular dystrophy. Neuromuscul Disord.

[45] Ito K, Takakusa H, Kakuta M (2021). Renadirsen, a Novel 2’OMeRNA/ENA® Chimera Antisense Oligonucleotide, Induces Robust Exon 45 Skipping for Dystrophin In Vivo. Curr Issues Mol Biol.

[46] Goyenvalle A, Griffith G, Babbs A (2015). Functional correction in mouse models of muscular dystrophy using exon-skipping tricyclo-DNA oligomers. Nat Med.

[47] Zarrouki F, Relizani K, Bizot F (2022). Partial Restoration of Brain Dystrophin and Behavioral Deficits by Exon Skipping in the Muscular Dystrophy X-Linked (mdx) Mouse. Ann Neurol.

[48] Tsoumpra MK, Fukumoto S, Matsumoto T (2019). Peptide-conjugate antisense based splice-correction for Duchenne muscular dystrophy and other neuromuscular diseases. EBioMedicine.

[49] Betts C, Saleh AF, Arzumanov AA (2012). Pip6-PMO, A New Generation of Peptide-oligonucleotide Conjugates With Improved Cardiac Exon Skipping Activity for DMD Treatment. Mol Ther Nucleic Acids.

[50] McClorey G, Banerjee S (2018). Cell-Penetrating Peptides to Enhance Delivery of Oligonucleotide-Based Therapeutics. Biomedicines.

[51] Sarepta Therapeutics Announces Positive Data from Part B of MOMENTUM, a Phase 2 Study of SRP-5051 in Patients with Duchenne Muscular Dystrophy Amenable to Skipping Exon 51.

[52] Community Letter: Update SRP-5051 Program.

[53] Sarepta Therapeutics Announces Third Quarter 2024 Financial Results and Recent Corporate Developments.

[54] Larkindale J, Lonkar P, Goyal J (2023). P44 Phase 1 study of PGN-EDO51 demonstrates tolerability, delivery and high levels of exon skipping for treatment of Duchenne muscular dystrophy (DMD). Neuromuscular Disorders.

[55] PepGen Reports Positive Data from Phase 1 Trial of PGN-EDO51 for the Treatment of Duchenne Muscular Dystrophy.

[56] Yin H, Saleh AF, Betts C (2011). Pip5 transduction peptides direct high efficiency oligonucleotide-mediated dystrophin exon skipping in heart and phenotypic correction in mdx mice. Mol Ther.

[57] Betts CA, Saleh AF, Carr CA (2015). Prevention of exercised induced cardiomyopathy following Pip-PMO treatment in dystrophic mdx mice. Sci Rep.

[58] Chwalenia K, Oieni J, Zemła J (2022). Exon skipping induces uniform dystrophin rescue with dose-dependent restoration of serum miRNA biomarkers and muscle biophysical properties. Mol Ther Nucleic Acids.

[59] Entrada Therapeutics | Entrada Therapeutics Reports Positive Preliminary Data in Healthy Volunteers from Phase 1 ENTR-601-44-101 Trial for Duchenne Muscular Dystrophy.

[60] Avidity Biosciences Reports Positive Data Demonstrating AOC 1044 Delivers Unprecedented Concentrations of PMO in Muscle Following a Single Dose in Healthy Volunteers from Phase 1/2 EXPLORE44™ Trial for Duchenne Muscular Dystrophy.

[61] Desjardins CA, Yao M, Hall J (2022). Enhanced exon skipping and prolonged dystrophin restoration achieved by TfR1-targeted delivery of antisense oligonucleotide using FORCE conjugation in mdx mice. Nucleic Acids Res.

[62] Dyne Reports Positive Phase 1/2 Data for Duchenne Agent DYNE-251.

[63] Dyne Therapeutics Presents Data at World Muscle Society Congress Highlighting Promise of FORCE™ Platform to Address Underlying Causes of Neuromuscular Diseases.

[64] Ideker T, Thorsson V, Ranish JA (2001). Integrated genomic and proteomic analyses of a systematically perturbed metabolic network. Science.

[65] Roberts TC, Johansson HJ, McClorey G (2015). Multi-level omics analysis in a murine model of dystrophin loss and therapeutic restoration. Hum Mol Genet.

[66] Hoy SM (2023). Delandistrogene Moxeparvovec: First Approval. Drugs.

[67] Mendell JR, Shieh PB, McDonald CM (2023). Expression of SRP-9001 dystrophin and stabilization of motor function up to 2 years post-treatment with delandistrogene moxeparvovec gene therapy in individuals with Duchenne muscular dystrophy. Front Cell Dev Biol.

[68] First Clinical Trial Results of Gene Therapy (GNT0004) for Duchenne Muscular Dystrophy presented at International Myology 2024 Congress.

[69] (2020). High-dose AAV gene therapy deaths. Nat Biotechnol.

[70] Tinsley JM, Fairclough RJ, Storer R (2011). Daily Treatment with SMTC1100, a Novel Small Molecule Utrophin Upregulator, Dramatically Reduces the Dystrophic Symptoms in the mdx Mouse. PLoS One.

[71] Tabebordbar M, Zhu K, Cheng JKW (2016). In vivo gene editing in dystrophic mouse muscle and muscle stem cells. Science (1979).

[72] Hanson B, Stenler S, Ahlskog N (2022). Non-uniform dystrophin re-expression after CRISPR-mediated exon excision in the dystrophin/utrophin double-knockout mouse model of DMD. Mol Ther Nucleic Acids.

[73] Long C, Amoasii L, Mireault AA (2016). Postnatal genome editing partially restores dystrophin expression in a mouse model of muscular dystrophy. Science (1979).

[74] van Westering TLE, Lomonosova Y, Coenen-Stass AML (2020). Uniform sarcolemmal dystrophin expression is required to prevent extracellular microRNA release and improve dystrophic pathology. J Cachexia Sarcopenia Muscle.

[75] Morin A, Stantzou A, Petrova ON (2023). Dystrophin myonuclear domain restoration governs treatment efficacy in dystrophic muscle. Proc Natl Acad Sci U S A.

[76] Morgan JE, Hoffman EP, Partridge TA (1990). Normal myogenic cells from newborn mice restore normal histology to degenerating muscles of the mdx mouse. J Cell Biol.

[77] Morgan JE, Coulton GR, Partridge TA (1989). Mdx muscle grafts retain the mdx phenotype in normal hosts. Muscle Nerve.

[78] Watt DJ, Morgan JE, Clifford MA (1987). The movement of muscle precursor cells between adjacent regenerating muscles in the mouse. Anat Embryol (Berl).

[79] Partridge TA, Morgan JE, Coulton GR (1989). Conversion of mdx myofibres from dystrophin-negative to-positive by injection of normal myoblasts. Nature.

[80] Boldrin L, Zammit PS, Morgan JE (2015). Satellite cells from dystrophic muscle retain regenerative capacity. Stem Cell Res.

[81] Rossi R, Singh S, Torelli S (2023). P29 DMD transcript imbalance and nuclear trafficking evaluation in muscle biopsies from baseline and golodirsen treated 4053-101 clinical trial patients. Neuromuscular Disorders.

[82] Anthony K, Feng L, Arechavala-Gomeza V (2012). Exon skipping quantification by quantitative reverse-transcription polymerase chain reaction in Duchenne muscular dystrophy patients treated with the antisense oligomer eteplirsen. Hum Gene Ther Methods.

[83] Morgan JE, Beauchamp JR, Pagel CN (1994). Myogenic Cell Lines Derived from Transgenic Mice Carrying a Thermolabile T Antigen: A Model System for the Derivation of Tissue-Specific and Mutation-Specific Cell Lines. Dev Biol.

